# Basilar artery diameter as neuroimaging biomarker in Chinese Fabry disease patients

**DOI:** 10.1186/s13023-023-02759-6

**Published:** 2023-07-10

**Authors:** Yan Lok Tiffany Lam, Bun Sheng, Hoi Ming Kwok, Ellen Lok Man Yu, Ka Fai Johnny Ma

**Affiliations:** 1 Department of Medicine & Geriatrics, Princess Margaret Hospital, 2-10 Princess Margaret Hospital Road, Lai Chi Kok, Hong Kong Special Administrative Region Hong Kong; 2 Department of Diagnostic and Interventional Radiology, Princess Margaret Hospital, Hong Kong Special Administrative Region, Lai Chi Kok, Hong Kong; 3 Clinical Research Centre, Kowloon West Cluster, Hong Kong Special Administrative Region, Kowloon, Hong Kong

**Keywords:** Fabry disease, Basilar artery, Vertebrobasilar dolichoectasia, IVS4, Chinese

## Abstract

**Background:**

Fabry disease (FD) is an X-linked lysosomal storage disease resulting from mutations of α-galactosidase A gene, and has been emphasized as one of the etiologies of young stroke and leukoencephalopathy. Vertebrobasilar dolichoectasia (VBD) is a highlighted finding in FD. We aim to examine the utility of VBD in Chinese FD by comparing the differences in basilar artery (BA) diameter of Chinese FD patients against age-matched controls with and without stroke.

**Methods:**

This was a matched case-control study involving 37 Chinese FD patients. The BA diameters were evaluated on axial T2-weighted magnetic resonance imaging and compared to two age-and-gender matched control groups, one with stroke and one without. The association between BA diameter and stroke occurrences and white matter hyperintensities (WMH) were analyzed among all FD patients.

**Results:**

Patients with FD had significantly increased BA diameter compared to controls with and without stroke (p < 0.001). A BA diameter of 4.16 mm could distinguish FD from controls in the stroke subgroup (ROC AUC 0.870, p = 0.001, sensitivity 80% specificity 100%), and with a cut-off of 3.21 mm in the non-stroke subgroup (ROC AUC 0.846, p < 0.001, sensitivity 77.8% specificity 88.9%). Larger BA diameter had more stroke occurrences and was moderately associated with heavier WMH load in terms of higher total FAZEKAS scores. (Spearman’s rho = 0.423, p = 0.011).

**Conclusion:**

VBD was also present in Chinese FD patients. BA diameter has high diagnostic utility in identifying FD from a mixed cohort of stroke and normal controls, and carried predictive value in evaluating neurological complications of FD.

## Background

Fabry disease (FD) is an X-linked lysosomal storage disease resulting from mutations of α-galactosidase A (GLA) gene. The deficiency in GLA enzyme activity leads to accumulation of globotriaosylceramide (Gb3) and glycosphingolipids deposition causing cellular dysfunction, ultimately resulting in multisystem disorders [1]. The neurological hallmarks of Fabry disease include small fiber neuropathy, cerebral microangiopathy and macroangiopathy such as progressive white matter lesions or even devastating stroke [2]. 70% of Fabry-stroke patients had stroke as their first major disease-specific complication, and it estimated to contribute to 1% of young stroke and 4.5% of young male cryptogenic stroke in the general population [3, 4].

Basilar artery (BA) diameter has been found significantly enlarged in FD patients when compared to healthy controls of the same age [5–7]. Excessive lipid deposition in the abnormal smooth muscle vacuoles trigger inflammatory process of arterial wall intima, leading to luminal stenosis and weakening of arterial wall microvasculature, resulting in tortuosity, elongation, and dilatation of the vessels [8]. It is postulated that BA is predominantly affected because the dynamic cerebral autoregulation is more vulnerable with comparatively less sympathetic innervation of intracranial arterioles in the posterior cerebral circulation. Vertebrobasilar dolichoectasia (VBD) is independently associated with small vessel vasculopathy, strokes, and increased cerebrovascular and cardiovascular morbidity and mortality [9–12].

There is increasing awareness of FD in Southern China after the identification of a highly prevalent later-onset cardiac variant c.640-801G > A at intron 4 (IVS4) in the region, which is not seen in other ethnic populations [13]. It is unknown whether the VBD associated with FD also exists in Chinese FD. In this study, we explore the BA diameter and its association with CNS manifestation, mainly cerebral white matter hyperintensities (WMH) and stroke occurrences, in a cohort of Chinese FD patients treated in Hong Kong. We also examine the diagnostic utility of BA diameter in FD by quantitatively measuring the BA diameter and compare it with healthy controls and stroke patients in our locality.

## Materials and methods

### Subjects

We conducted a matched case-control study in Princess Margaret Hospital, which was the designated FD treatment centre in Hong Kong. This study was approved by Kowloon West Cluster research ethics committee [EX-21-097(160 − 13)]. We enrolled 37 Chinese FD patients who were diagnosed by GLA sequencing, enzyme assay, and/or histology. The demographic data, medical history, investigation results and MRI films were retrieved from electronic patient records and laboratory database.

Two control groups were selected in a 1:2 ratio, matched to gender and age ± 5 years, to compare to FD with and without stroke respectively. The first group was stroke patients discharged from acute stroke unit and second group was individuals who had MRI brain performed in Princess Margaret Hospital between 2019 and 2020.

We also excluded patients who were non-Chinese ethnicity, had neurosurgery, cryptogenic stroke, end-stage renal failure, polycystic kidney disease, or poorly controlled hypertension (on > 2 anti-hypertensive drugs), fetal origin of posterior cerebral artery, or posterior cranial fossa pathologies that might alter the course of BA, such as neoplasm, vascular malformation, demyelination, and brain abscess etc.

### Neuroimaging evaluation

The measurements of BA diameter and WMH were obtained from each individual MRI imaging. The BA diameter was defined by its short axis diameter as measured at the mid-pons level on axial T2-weighted MRI sequence. Two FD patients who had stroke, but no brain MRI imaging had their BA diameter evaluated via axial CT scans. All the neuroimagings were independently evaluated by the author (YLT Lam) and a radiologist (HM Kwok) blinded to the clinical information. The inter-rater reliability was validated. When the ratings differed between observers, the average measurement of BA diameter, rounded up to the nearest 0.01mm, was used.

To determine the burden of WMH, we semi-quantitatively graded the WMH load by FAZEKAS grading on T2-weighted and fluid-attenuated inversion recovery (FLAIR) sequences on MRI scans. Each MRI scan was assessed for the presence and extent of white matter hyperintensity by periventricular hyperintensities (PVH) and deep white matter hyperintensities (DWMH). PVH was classified by grade 0, absent; grade 1, pencil-like thin linings; grade 2, smooth haloes; grade 3, irregular periventricular signal extending to deep white matter. DWMH was classified by grade 0, absent; grade 1, punctate separate foci; grade 2, fusing lesions, grade 3, large confluent areas.

### Statistical analysis

All data analysis was computed using IBM SPSS statistical software version 26.0. We used sample t-tests to compare the BA diameter, chi-square test and Fisher’s exact test for group comparisons of categorical variables and Mann-Whitney U tests for non-parametric unrelated data comparison. Spearman’s rho correlation test was applied to quantify the association between BA diameter and FAZEKAS score. Receiver operating characteristic curves (ROC) and unweighted Youden method were adopted to define the BA diameter cut-off value, sensitivity and specificity. The area under the ROC curve (AUC) was calculated with corresponding 95% confidence interval (95% CI). Descriptive statistics were presented as mean (SD) and median (IQR) where appropriate. All the statistics were two-end conducted at 0.05 level of significance. The inter-rater reliability of BA diameter analyses was determined by the intraclass coefficient correlation.

## Results

### Baseline demographics of Fabry & non-fabry group

Demographics and clinical characteristics of the 37 FD patients were presented in Table 1. The average age was 56.8±11.6 years old and 73% were male. Among the male FD patients, the mean age was 61 (ranging 20 to 72 years old), and there were 10 female FD patients with mean age of 52 (ranging 42 to 66 years old). Ten (27%) suffered from stroke, all had cerebral infarctions and microbleeds were found in three of these patients. One patient had traumatic subdural hematoma. The average age of those who suffered from stroke were older, with an average age of 61.4±10 years old. Despite that, there were no significant differences among FD and the two control groups in terms of age, gender, smoking and drinking history. Proteinuria and left ventricular hypertrophy were statistically increased in FD group when compared with both matched control groups with and without history of stroke. Other medical comorbidities including hypertension, diabetes, chronic kidney disease, coronary artery disease and cardiac arrhythmia were similar for all groups. More than half (54.1%, 75% male) of the FD patients were on enzyme replacement therapy (ERT), in which one who was on treatment suffered from stroke. GLA sequencing was performed in all FD patients. Four patients (10.8%) had classical FD genotypes, seventeen (45.9%) had IVS4 mutations, and the remaining were other missense mutations.

**Table 1:** Baseline demographics and characteristics of Fabry patients, patients with stroke, and normal controls

	All Fabry (N = 37)		Matched for stroke					Matched for non-stroke				
			Fabry (N = 10)		Control (N = 20)		p value	Fabry (N = 27)		Control (N = 54)		p value
Age, y, mean ± SD	56.8 ± 11.6		61.4 ± 10.0		61.3 ± 10.3		NS^†^	55.1 ± 11.9		55.1 ± 11.8		NS
Male, n (%)	27 (73.0)		8 (80)		16 (80)		NS	19 (70.4)		38 (70.4)		NS
Chronic or history of smoking, n (%)	10 (27.0)		4 (40)		9 (45)		NS	6 (22.2)		13 (24.1)		NS
Chronic drinking, n (%)	6 (16.2)		0		5 (25)		NS	6 (22.2)		7 (13.0)		NS
BA diameter, mm, mean ± SD	3.9 ± 1.0		4.5 ± 1.1		3.2 ± 0.6		< 0.001	3.7 ± 0.9		2.8 ± 0.4		< 0.001
WMH Fazekas score^‡^, median [IQR]	2 [1–3]		4 [2–4]		—		—	2 [1–2]		—		—
0–2	24 (68.6)		3 (37.5)		—			21 (77.8)		—		
3–4	9 (25.7)		4 (50)		—			5 (18.5)		—		
5–6	2 (5.7)		1 (12.5)		—			1 (3.7)		—		
ERT, n (%)	20 (54.1)		1 (10)		—		—	19 (70.4)		—		—
Hemorrhage/microbleeds, n (%)			4 (40)		—		—	—		—		
Area of infarction, n (%)							NS					—
AC	—		5 (50)		10 (50)			—		—		
PC	—		3 (30)		5 (25)			—		—		
Both	—		2 (20)		5 (25)			—		—		
Hypertension, n (%)	20 (54.1)		7 (70)		17 (45.9)		NS	13 (48.1)		10 (18.5)		0.005
Diabetes mellitus, n (%)	4 (10.8)		3 (30)		3 (8.1)		NS	1 (3.7)		8 (14.8)		NS
eGFR (mL/min/1.73m2), mean ± SD	70.0 ± 21.2		70.3 ± 19.4		75.5 ± 18.2		NS	69.9 ± 22.2		84.1 ± 11.8		< 0.001
Proteinuria, n (%)	16 (43.2)		5 (50)		1 (5)		0.009	11 (40.7)		1 (1.9)		< 0.001
Chronic kidney disease^§^, n (%)	12 (32.4)		4 (40)		3 (15)		NS	8 (29.6)		7 (13.0)		NS
RRT/Transplantation, n (%)	7 (18.9)		3 (30)		1 (5)		NS	4 (14.8)		0		0.011
Cardiomyopathy, n (%)	24 (64.9)		8 (80)		1 (5)		< 0.001	16 (59.3)		0		< 0.001
Coronary artery disease, n (%)	4 (10.8)		2 (20)		2 (10)		NS	2 (7.4)		2 (3.7)		NS
Cardiac arrhythmia, n (%)	8 (21.6)		5 (50)		3 (15)		NS	3 (11.1)		2 (3.7)		NS
Positive family history before diagnosis	14 (37.8)		5 (25)		—			9 (33.3)		—		—

^†^ NS = not reaching statistical significance

^‡^ 2 missing observations

^§^ eGFR < 60 mL/min/1.73 m2

BA = basilar artery; WMH = white matter hyperintensity; ERT = enzyme replacement therapy; AC = Anterior circulation; PC = posterior circulation; RRT = renal replacement therapy

### BA diameter

**Fig. 1:**
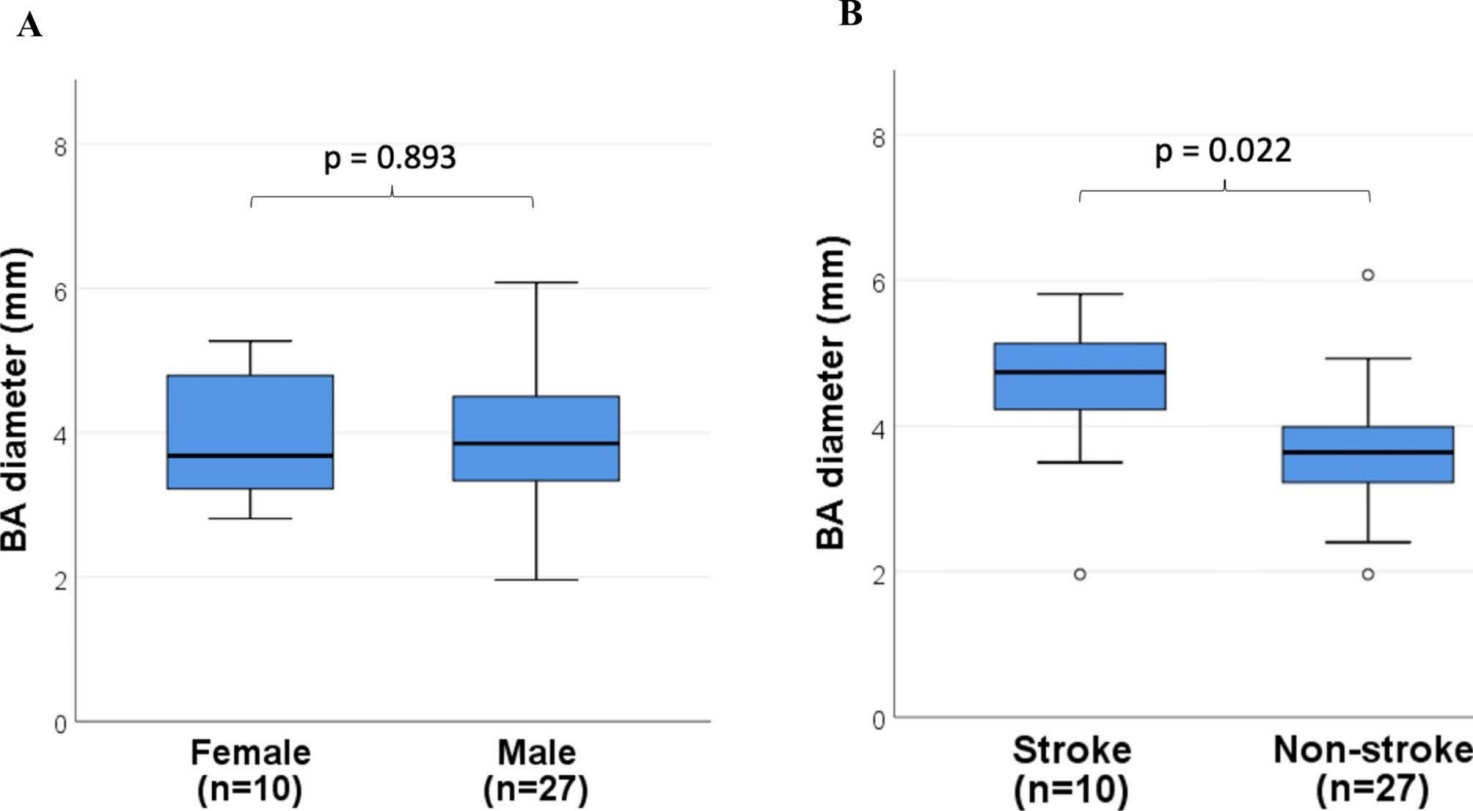
Differences in BA diameter among FD patients. **(A)** Boxplots showing no significant BA diameter differences between genders. **(B)** Larger BA diameter in FD stroke patients compared with those without stroke. **(C)** No significant BA diameter differences between classical and non-classical genotypes

**Fig. 2:**
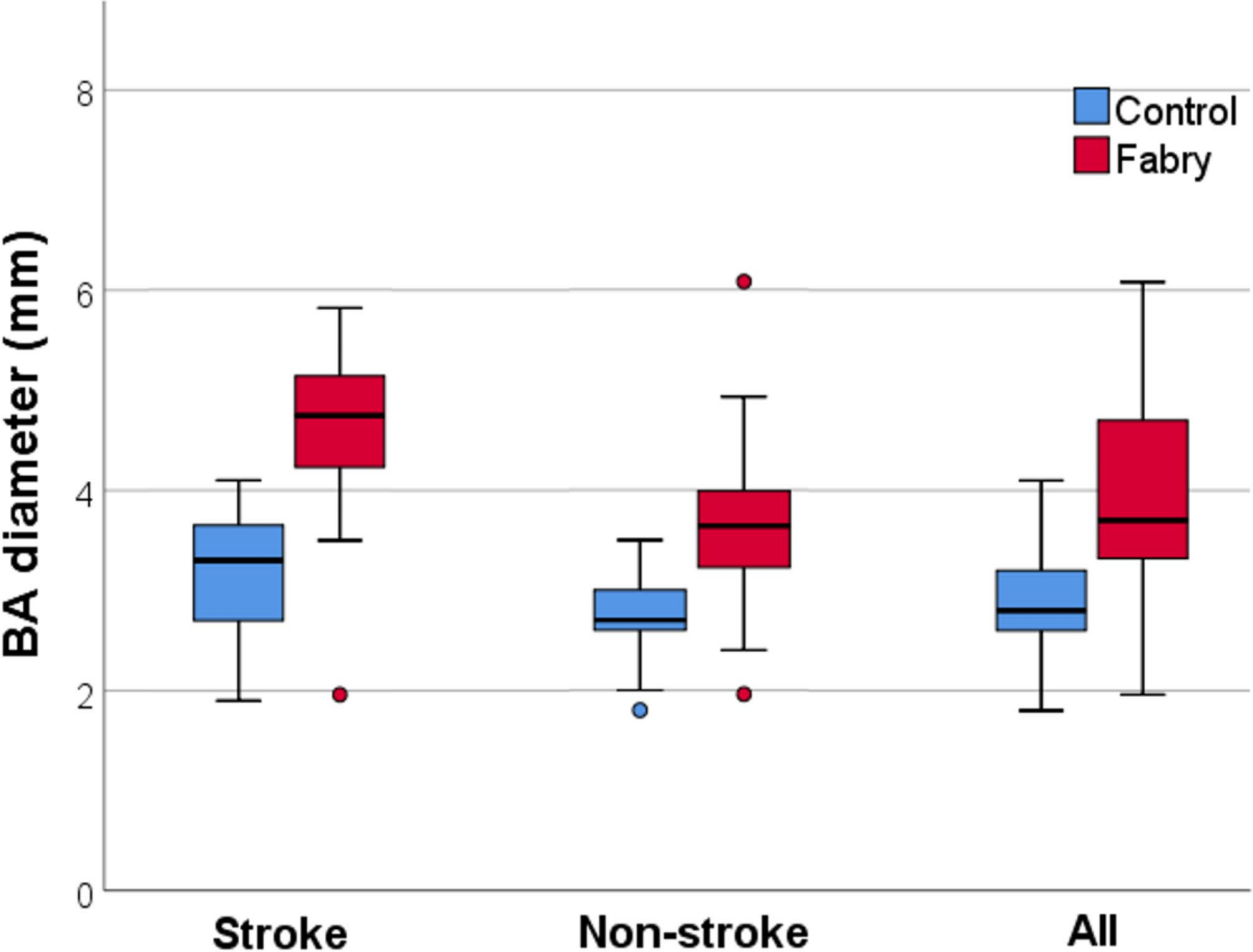
Comparison of BA diameter. Significantly enlarged BA diameter was observed when comparing FD patients against controls in both stroke and non-stroke subgroups. (p < 0.001)

The mean BA diameter of all FD patients was 3.9 mm±1.0 (3.9 mm±1.1 for male FD patients and 3.68 mm±0.8 for female FD patients). BA diameters did not differ significantly between male and female among the FD group. [Fig. 1a]. For those who suffered from stroke, the mean BA diameter was 4.5 mm±1.1, and those who did not had a mean BA diameter of 3.7 mm±0.9. [Fig. 1b] Both groups had significantly enlarged BA diameter when compared to the gender and age-matched control groups. [Fig. 2] The mean BA diameter among IVS4 FD patients was 3.9 mm±1.2. Comparison of BA diameter between classical and non-classical genotype of FD was insignificant (3.88 mm±0.6 for classical FD and 3.82 mm±1.1 for non-classical FD). [Fig. 1c]

**Fig. 3:**
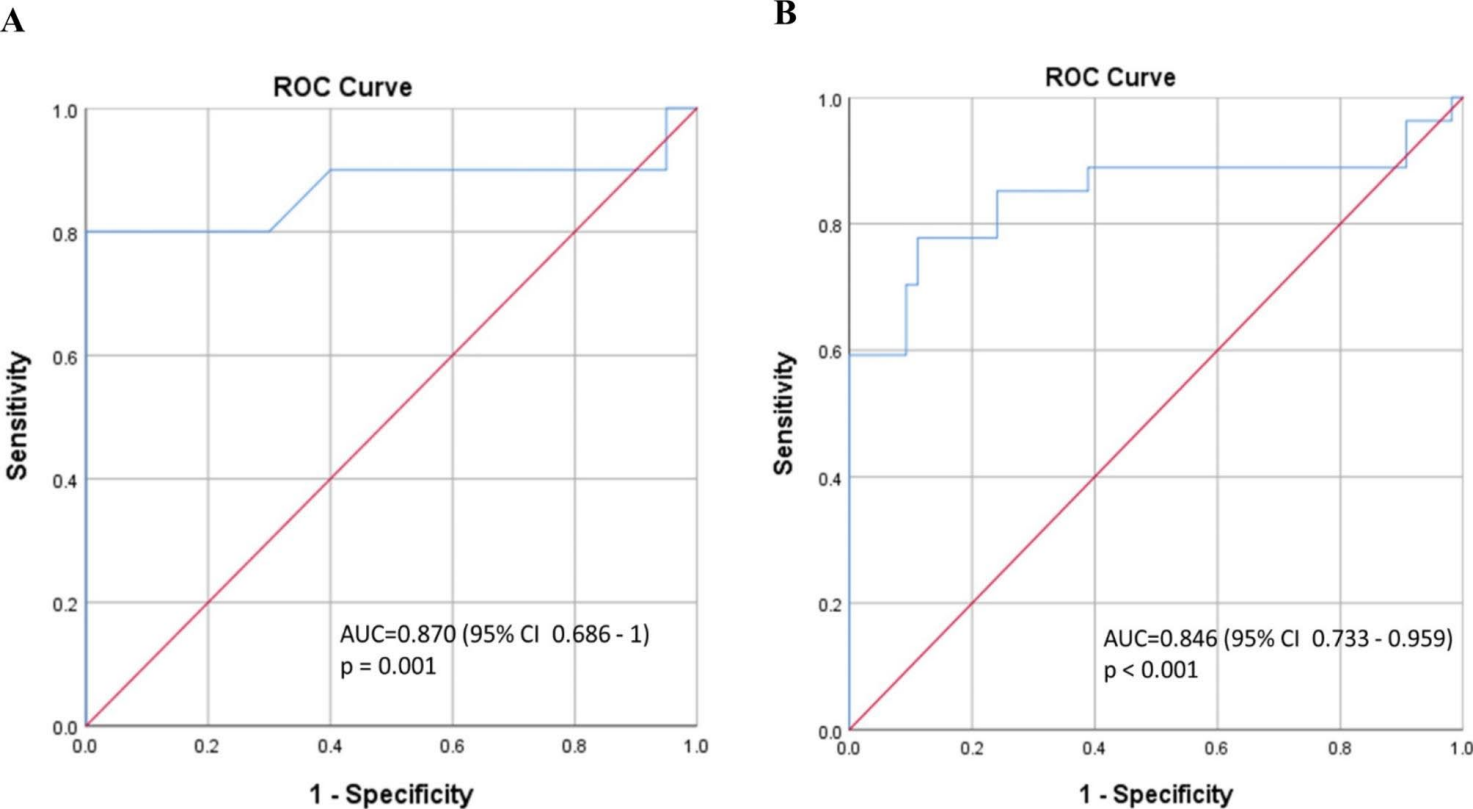
Receiver operating curves (ROC) analysis of BA diameters. ROC data showing high sensitivity and specificity of BA diameter comparing **(A)** FD patients (N = 10) versus controls (N = 20) in stroke subgroup. **(B)** FD patients (N = 27) versus controls (N = 54) in non-stroke subgroup

From the ROC analysis of BA diameter, BA diameter could distinguish Fabry from control in both stroke and non-stroke subgroups. [Fig. 3] The AUC comparing Fabry who suffered from stroke against control stroke groups was 0.870 (95% CI 0.686-1), p = 0.001, while that in non-stroke subgroup was 0.846 (95% CI 0.733–0.959), p < 0.001. Taking the BA diameter cut-off value of 4.16 mm, it could safely differentiate Fabry patient with stroke from control group with a sensitivity of 80% and specificity of 100%. Whereas Fabry without stroke could be separated from non-stroke patients with a BA diameter of 3.21 mm (sensitivity 77.8%, specificity 88.9%).

Regarding the interrater reliability of MRI-measured BA diameters, the intraclass correlation coefficient was 0.92 (95% CI 0.85 to 0.96). The mean differences of BA diameter between both interpreters were 0.12 and there was no statistically significant discordance (p = 0.21). 89.2% of all discrepancies lie between − 0.8 and 0.8 mm without detectable systemic pattern.

### BA diameter, stroke and WMH in FD

#### Bigger BA diameter has more stroke occurrence

Among the ten FD patients who suffered from stroke, the BA diameter ranged from 2 to 5.5 mm. Two patients did not have MRI brain performed, thus CT measurements were used. Those who suffered from stroke had a larger mean BA diameter (4.5 mm±1.1) than those who did not have stroke (3.7 mm±0.9). [Fig. 1b]

#### FD patients with stroke

**Fig. 4:**
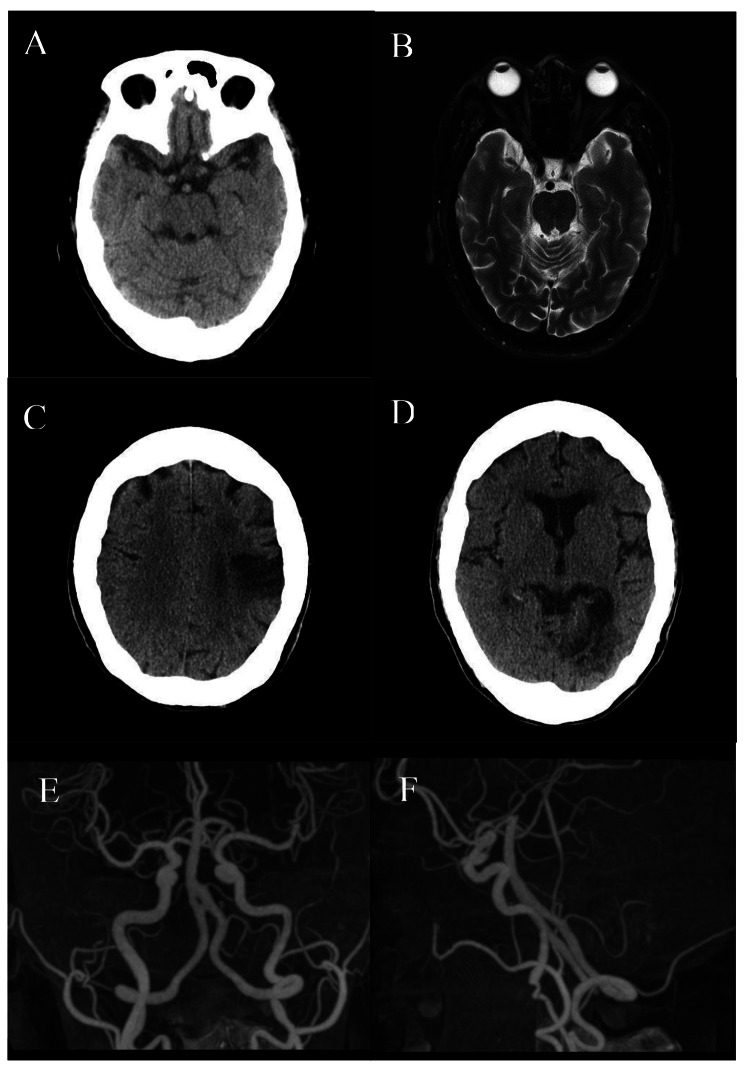
66-year-old FD female patient who suffered from recurrent ischaemic strokes. **(A)** Initial CT Brain showing prominent BA. **(B)** BA diameter measuring 5.3mm on axial T2-weighted MRI imaging. Recurrent ischaemic strokes **(C)** left parietal infarct in left middle cerebral artery territory **(D)** left occipital infarct in left posterior cerebral artery territory. Intracranial arterial dolichoectasia was appreciated on CT angiography in PA view **(E)** and lateral view **(F)**. Both anterior and posterior vessels were elongated, tortuous and ecstatic

There were ten patients (aged 61.4±10, 80% male) who suffered from stroke among the cohort of FD patients, all of which suffered from ischaemic stroke. One female FD patient suffered repeated episodes of ischaemic strokes at the age of 66 and 67. Her neuroimagings were presented in Fig. 4. One of the male patients had classical FD, while five carried the IVS4 mutation. Both anterior and posterior circulation territories, as well as cortical and subcortical stroke locations were involved. The anterior vascular territory of ischaemic stroke was predominantly involved in our cohort, and the proportion of stroke locations was comparable with the control group. Other demographics and details of stroke characteristics of our cohort of FD patients were listed in Table 2.

**Table 2:** Characteristics of FD patients with stroke

Gender	Age of Stroke	Smoker	Genotype	GLA Activity	Lyso-Gb3 level (ng/mL)	ERT	BA Diameter (mm)	Fazekas Score	Stroke Details	Other Medical Conditions
M	68	+ (Ex)	c.640-801G > A	0.54µmol/L wb/hr	NA	⎯	5.5 (CT)	NA	(1) Bilateral BG, CR, L thalamus lacunar infarcts (2) Traumatic SDH	HT, DM, CHB on PPM, cardiomyopathy, IHD, NSVT, AF with LAAO
M	69	⎯	c.640-801G > A	NA	NA	⎯	4.1	2	(1) Bilateral lacunar infarcts (2) R frontal hemosiderin deposits	HT, DM, HL, CHB, IHD with PCI, polymorphic VT on ICD; DLBCL
F	66,67	⎯	c.346G > T	2.07µmol/L/h	8.568	⎯	5.3	4	(1) Cortical stroke L MCA territory (2) R lacunar infarct (3) Multiple old microbleeds	AF, cardiomyopathy, dementia, bipolar affective disorder
M	47	+ (Ex)	c.803_806delTAGT^†^	0.29 nmol/mg prot/hr	NA	⎯	4.7	4	(1) R IC and L parietal lobe lacunar infarcts (2) Bilateral supratentorial microbleeds	ESRF with renal transplant, cardiomyopathy, pAF with cryoablation, peripheral neuropathy, polycythaemia
M	53	⎯	c.346G > A	0.17µmol/h	43.48	⎯	5.3	5	(1) R thalamic lacunar infarct (2) R cerebellar infarct	ESRF with renal transplant, AF, cardiomyopathy, AVN
M	72	+ (Ex)	c.640-801G > A	1.37µmol/h	NA	⎯	4.7 (CT)	NA	Bilateral BG lacunar infarcts	ESRF (IgA) with renal transplant, HT, cardiomyopathy (HCM with alcohol septal ablation and DDDR pacemaker)
F	43	⎯	c.145C > G	2.64µmol/h	2.99	⎯	5	1	R thalamic infarct	HT, IFG, HL, proteinuria, CA breast
M	67	⎯	c.640-801G > A	1.23µmol/h	5.74	⎯	2	2	L BG infarct	HT, DM, HL, cardiomyopathy
M	64	⎯	c.109G > A	0.26µmol/h	27.5	+	4.7	4	Bilateral centrum semiovale infarct	HL, cardiomyopathy, nephropathy (IgAN + Fabry)
M	65	+ (Ex)	c.640-801G > A	1.08µmol/L/h	5.18	⎯	3.5	4	L cerebellum and L parietal infarct	HT, HL, Gout, cardiomyopathy

^†^Classical phenotype

GLA = alpha-galactosidase A; lyso-Gb3 = globotriaosylsphingosine; ERT = enzyme replacement therapy; BA = basilar artery; BG = basal ganglia; CR = corona radiata; SDH = subdural hematoma; MCA = middle cerebral artery; IC = internal capsule; L = left; R-right; HT = hypertension; DM = diabetes mellitus; HL = hyperlipidemia; IFG = impaired fasting glucose; CHB = complete heart block; IHD = ischaemic heart disease; NSVT = non-sustained supraventricular tachycardia; AF = atrial fibrillation; LAAO = left atrial appendage occlusion; ICD = implantable cardiac defibrillator; HCM = hypertrophic cardiomyopathy; DLBCL = diffuse large B cell lymphoma; ESRF = end stage renal failure; AVN = avascular necrosis

#### Larger BA diameter has higher WMH load

**Fig. 5:**
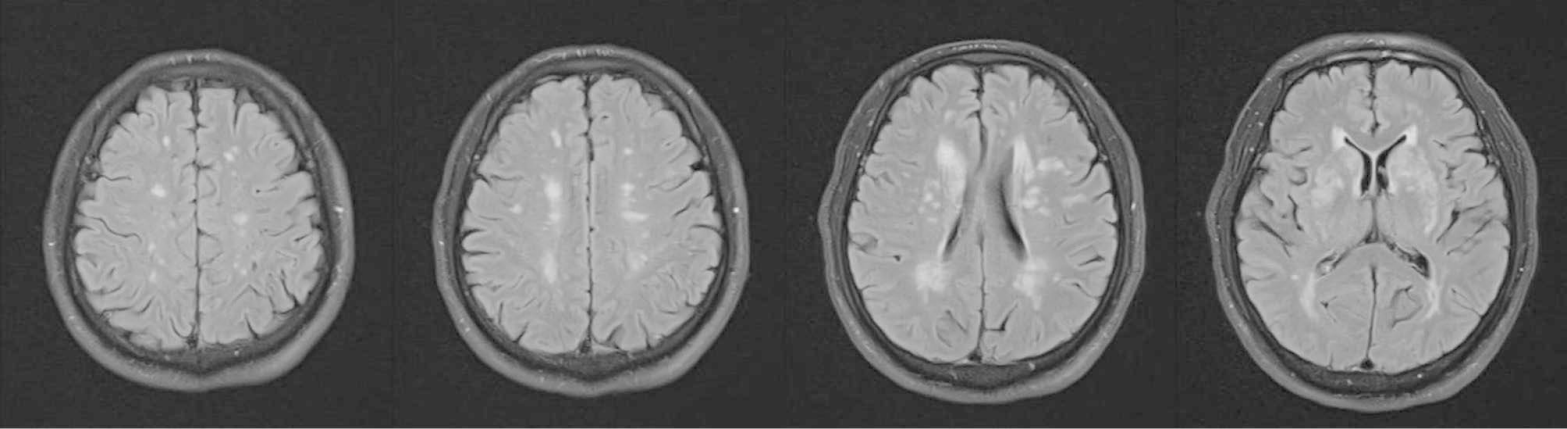
A 54-year-old IVS4 FD patient without hypertension suffering from advanced FD leukoencephalopathy. Serial axial FLAIR images demonstrated heavy white matter burden at periventricular and deep white matter regions. Large areas of patchy and confluent T2/FLAIR hyperintense signals were appreciated

**Fig. 6:**
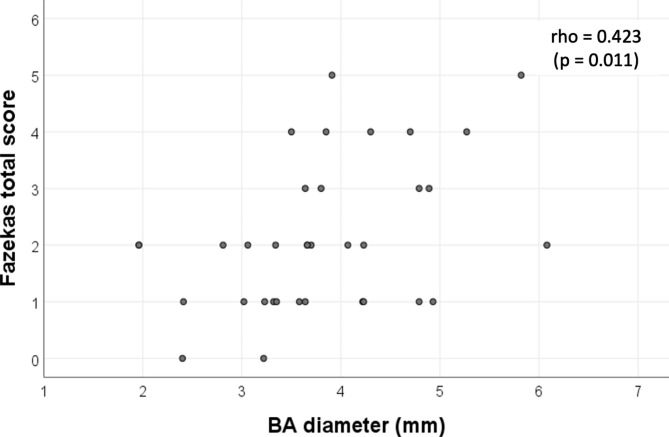
Relationship between BA diameter and CWML burden in FAZEKAS visual rating score. Scatter plot graph showing moderate correlation between BA diameter and FAZEKAS total score (Spearman’s rho correlation 0.423, p = 0.011). Larger BA diameter was associated with higher WML burden and leukoencephalopathy

The median total WMH FAZEKAS score of all FD patients was 2 [IQR 1–3]. Most of our FD patients had a low FAZEKAS score. Reasonably those with stroke had a higher median total score (4 [IQR 2–4]) than those who did not (2 [IQR 1–2]). Example of a FD patients with high FAZEKAS score was illustrated in Fig. 5. Significant correlation between BA diameter and WMH was observed. Larger BA diameter was moderately associated with higher total FAZEKAS score, reflecting greater WMH load. (Spearman rho = 0.423, p = 0.011). [Fig. 6] None of our patients had pulvinar hyperintensity on T1-weighted images.

## Discussion

FD is an increasingly recognized but still underdiagnosed lysosomal storage disorder. Those who has non-classical variants due to missense and splicing mutations often has milder disease severity and later onset of clinical manifestations due to higher residual alpha-galactosidase activity. 37.8% of our subjects are identified and diagnosed with FD through family cascade screening. Recognition of these later-onset FD patients is crucial as it would permit expeditious cascade screening and timely therapeutic intervention [14]. GLA enzyme assay is the recommended diagnostic test for male patients but it is less sensitive in female, whom would require GLA gene sequencing to make the diagnosis. Both tests, however, are not readily accessible in every institution. In this study, we explore the utility of BA diameter as an easily obtainable MRI biomarker for FD screening and prognosis.

This is the first study on VBD and FD in the Chinese population. We demonstrated that BA diameter was significantly enlarged compared to general population in both stroke and non-stroke subgroups. In a study conducted in Germany with 87 FD patients, investigators demonstrated a BA diameter > 3.2 mm could distinguish FD from normal controls with a high sensitivity of 87% and specificity of 86%, but it failed to find a difference in BA diameter between male FD patients and male stroke controls [5]. Another study has found a lower BA diameter cut-off of 2.98 mm for all FD patients versus non-FD stroke controls (sensitivity 84% specificity 88.5%) [7]. Since we had larger proportion of FD patients who suffered from stroke than other existing reports, we were able to conduct subgroup analysis on the predictive properties of BA diameter in both stroke and non-stroke categories. BA diameter of 4.16 mm has a very high sensitivity and specificity to distinguish our subgroup of FD from matched stroke controls. As for FD without stroke, we also found a significant BA diameter cut-off value of 3.21 mm with a sensitivity and specificity > 75%. These findings could assist physicians to decide whether to proceed for further FD diagnostic tests in stroke patients.

The BA cut-off values we obtained in our cohort of Chinese patients are bigger than the reported BA cut-off in other literature, probably due to inclusion of older FD patients especially patients with IVS4 variant. In fact, only 3 of our 10 FD stroke patients developed stroke at an age younger than 55. The five IVS4 patients who had stroke were between 65 and 72. Later-onset IVS4-type FD has long been considered as a cardiac variant, but studies in Taiwan have also shown that IVS4 patients have higher frequency of cerebral infarctions, pulvinar signs, and greater volume of WMH than general population [15]. Notably their BA diameter might even be larger than patients with classical FD [16]. We also compared BA diameter of FD classical genotypes against non-classical genotypes which included IVS4 and other missense mutations. The result was not significant but constrained by the limited number of classical genotypes of FD patients included in this cohort. Our study also demonstrated that FD patient with IVS4 mutation developed stroke at older age, they could be missed in most of the Fabry stroke screening programs where young stroke patients are targeted.

Though stroke is a common manifestation in FD, the stroke topography and exact stroke mechanism are unfortunately elusive. Among our ten patients who experienced stroke, each of them had more than one predisposing risk factors for stroke identified and the stroke etiology was diverse. Only one FD stroke patient did not have cardiomyopathy, while half of them had various degree of nephropathy. The proportion of cardiomyopathy among our cohort of Chinese FD patients was high, likely over-represented by the high prevalence of cardiac-variant IVS4 FD in our locality.

Another important finding of our study is the early onset of WMH among FD patients, in line with other existing data in which the surge of WMH burden started from the fourth decade and was earlier than the general population and patients with cerebrovascular diseases [17, 18]. The age of our cohort of Chinese FD patients was evenly distributed (eleven patients aged younger than 50, twelve patients aged between 50 and 64, and fourteen were at or above 65.) Only two patients did not have any WMH involvement, one-third (31.4%) had moderate CWML load with FAZEKAS score of at least 3. Among the young FD patients below 50, all patients had mild to moderate CWML involvement (FAZEKAS score 1–4). Particularly, our youngest patient also showed early WMH features at the age of 20, with punctate hyperintense foci present at the periventricular region. The two patients with the highest FAZEKAS score of 5 were in their fifties (aged 53 and 54). One of them was a IVS4 male patient who had serial MRIs performed. He already had mild bilateral microvascular ischaemic changes on his first MRI at the age of 46 and progressed to extensive white matter changes at the age of 54 despite treated with ERT and no known hypertension.

Although enzyme therapy becomes the standard of care in FD since 2001, its effect on cerebrovascular complications is seldom individually addressed. In a systematic review ERT appeared to decrease the stroke events, and another observational study from Japan suggested possible attenuation of BA diameter enlargement with ERT [19, 20]. Nevertheless, the data is not conclusive and further data from longitudinal study is required for a better understanding on the influence of disease modifying therapies such as ERT or chaperon therapy on BA diameter and stroke occurrence.

### Limitations

This study was limited by the retrospective design that the MRI technique was not standardized and we were unable to include the BA curved length, tortuosity index, laterality, and height of bifurcation in the measurements. We recommend full multimodal MRI and TOF-MRA imaging for better BA diameter measurements as BA sometimes extend obliquely and tortuously in which the largest cross-sectional diameter might not be observed at the mid-pons level.

## Conclusion

BA diameter was an easily reproducible screening tool with high diagnostic utility in identifying FD from a mixed cohort of stroke and normal controls in the Chinese population. We propose to screen for FD in Chinese stroke patients with a BA diameter larger than 4.16 mm. As BA diameter is positively correlated with higher stroke rate and WML load, all newly diagnosed FD patients are justified to obtain a MRI brain imaging as a baseline on cerebral FD involvement and future disease monitoring.

## Data Availability

The datasets used and/or analyzed during the current study are available from the corresponding author on reasonable request.

## Abbreviations

AUC: Area under the ROC curve
BA: Basilar artery
CI: Confidence interval
DWMH: Deep white matter hyperintensities
ERT: Enzyme replacement therapy
FD: Fabry disease
FLAIR: Fluid-attenuated inversion recovery
Gb3: Globotriaosylceramide
GLA: α-galactosidase A
IQR: Interquartile range
PVH: Periventricular hyperintensities
ROC: Receiver operating characteristic
SD: Standard deviation
VBD: Vertebrobasilar dolichoectasia
WMH: White matter hyperintensities
